# A Near-Miss Event: Constrictive Pericarditis Misdiagnosed as Heart Failure With Preserved Ejection Fraction

**DOI:** 10.7759/cureus.99563

**Published:** 2025-12-18

**Authors:** Karuna Rayamajhi, Fnu Parul, Majid Yavari, Kent Brummel, Christopher Scoma

**Affiliations:** 1 Internal Medicine, University of Michigan, Sparrow Hospital, East Lansing, USA; 2 Cardiology, University of Michigan, Ann Arbor, USA; 3 Radiology, University of Michigan, Ann Arbor, USA

**Keywords:** cardiac magnetic resonance imaging (cmri), chronic constrictive pericarditis, heart failure with preserved ejection fraction (hfpef), septal bounce, transthoracic echocardiography (tte), tte

## Abstract

Constrictive pericarditis (CP) is often misdiagnosed, making echocardiography essential for initial evaluation. We present a case of misdiagnosed constrictive pericarditis in a 66-year-old female with rheumatoid arthritis who presented with shortness of breath. A transthoracic echocardiogram (TTE) performed by a cardiologist initially reported diastolic dysfunction, leading to a diagnosis of heart failure with preserved ejection fraction (HFpEF), and she was discharged on diuretics. Despite adherence to therapy, she was readmitted multiple times for worsening symptoms over 18 months. A repeat TTE revealed ventricular septal bounce in early diastole and a positive annulus reversus sign, both of which were missed on the initial study. Ventricular septal bounce in early diastole reflects abnormal interventricular dependence due to a noncompliant, often thickened pericardium, which restricts diastolic filling and causes the septum to move abruptly with changes in intracardiac pressures, and the annulus reversus sign means that the medial (septal) mitral annular early diastolic velocity (e') exceeds the lateral e' which is a result of pericardial tethering of the lateral annulus and is a distinguishing feature of constrictive pericarditis, as opposed to restrictive cardiomyopathy, where both e' velocities are reduced but the normal relationship is preserved. Respiratory cycle monitoring and hepatic vein examination, critical for CP diagnosis, were not performed initially. Cardiac magnetic resonance imaging (MRI) was performed for the high suspicion of CP, which demonstrated pericardial thickening with septal bounce and interventricular dependence, confirming CP. The patient underwent pericardiectomy with full recovery of her symptoms. This case highlights the diagnostic challenge of CP, given its nonspecific symptoms, and underscores the importance of expert echocardiographic evaluation, multimodality imaging, and cardiac catheterization, which remains the gold standard, as CP can be easily overlooked.

## Introduction

Constrictive pericarditis (CP) is a rare diagnosis with an estimated prevalence of 0.2%-0.4% among patients who have undergone cardiac surgery or experienced pericardial trauma or inflammation [1]. It is a reversible condition in which granulation tissue forms either from chronic inflammation or fibrosis within the pericardium, resulting in a loss of pericardial elasticity and restricted ventricular filling, ultimately leading to diastolic heart failure. The types of CP are transient/subacute (weeks to months, potentially reversible), chronic (months to years, irreversible), and occult (clinically silent until provoked). The risk of developing constrictive pericarditis is highest following purulent or tuberculous pericarditis and much lower after idiopathic or viral pericarditis (<0.5%) [2,3]. Post-surgical and post-radiation cases are increasingly recognized due to advances in cardiac interventions and cancer therapies [4-6]. CP can also mimic heart failure with preserved ejection fraction (HFpEF), often presenting with symptoms of right-sided heart failure. It is frequently misdiagnosed, underscoring the importance of expert evaluation and the use of multimodality imaging.

This article was previously presented as a poster presentation at the 74^th^ Annual Scientific Session of the American College of Cardiology on March 31, 2025. Written informed consent for publication of this case report and all accompanying images was obtained from the patient.

## Case presentation

A 66-year-old female patient with a history of seropositive rheumatoid arthritis (RA) and hypertension presented with exertional dyspnea, orthopnea, and lower limb swelling for one and a half months. She denied chest pain, recent viral illness, or prior history of tuberculosis, radiation, or surgery. On presentation, her vital signs were stable (blood pressure (BP): 133/80 mmHg, heart rate (HR): 76 beats per minute (bpm), SpO₂: 96% on room air). A physical exam revealed bilateral pitting edema of the lower extremities and abdominal distension consistent with moderate ascites; chest examination was unremarkable.

**Figure 1:**
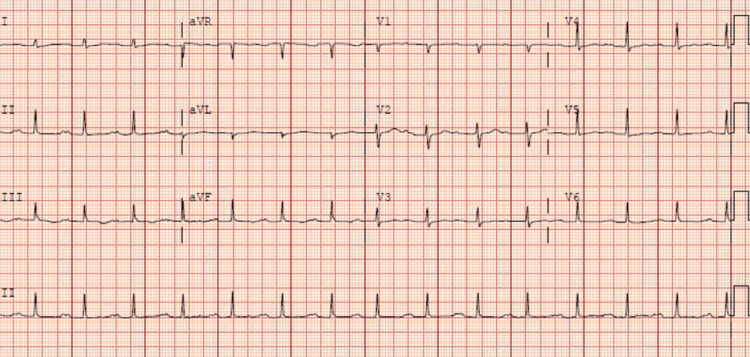
An electrocardiogram showed sinus rhythm, low voltage amplitude with nonspecific ST and T wave changes.

The electrocardiogram showed sinus rhythm with low-voltage QRS complexes and nonspecific ST-T wave changes (Figure 1).

**Figure 2:**
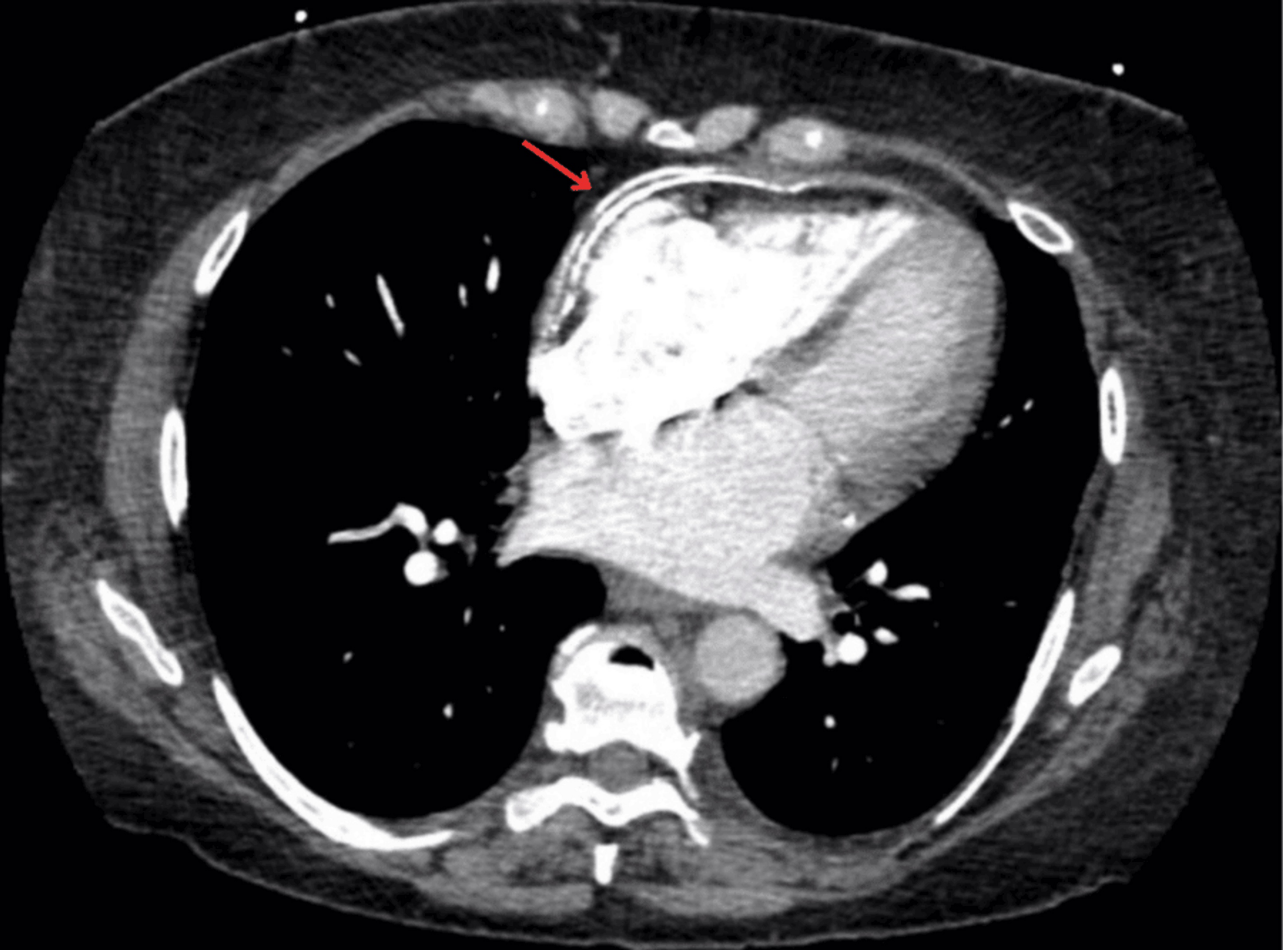
Computed tomography of the chest  showing extensive pericardial calcification, most severe along the right ventricular lateral wall, with small pericardial thickening/effusion with a maximum thickness of 7 mm.

Chest X-ray didn’t reveal any signs of constrictive pericarditis, but computed tomography (CT) performed 36 hours after presentation showed extensive pericardial calcification, most prominent along the lateral wall of the right ventricle, with mild pericardial thickening/effusion (maximum thickness: 7 mm; Figure 2).

**Figure 3:**
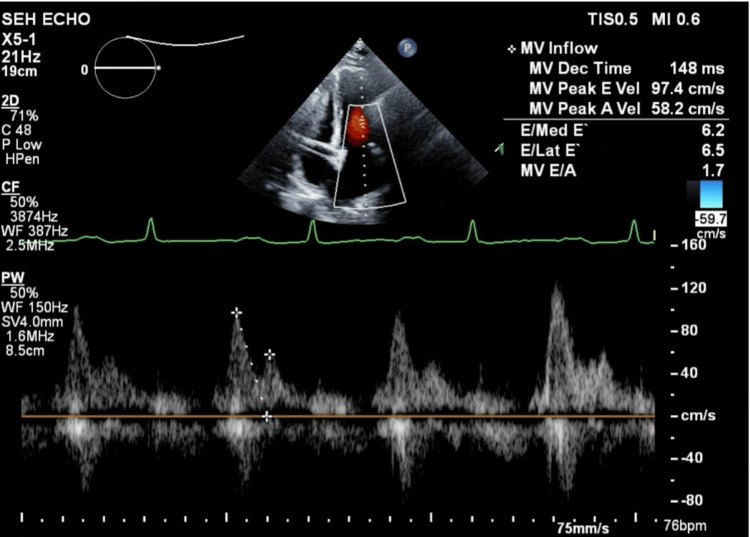
Transthoracic echocardiography showed preserved ejection fraction with mitral E/A ratio of 1.7 (greater than 1.5).

Laboratory evaluation showed a mildly elevated B-type natriuretic peptide (BNP) level of 225 pg/mL (reference range: 0-100 pg/mL); inflammatory markers were unremarkable; and a transthoracic echocardiogram (TTE) performed by a cardiologist revealed trivial pericardial effusion with preserved left ventricular volume and function (Figure 3), along with an elevated right ventricular systolic pressure (RVSP) of 46 mmHg.

She was subsequently diagnosed with HFpEF of 55%-60% and was discharged on diuretics, mineralocorticoid receptor antagonists, and sodium glucose co-transporter (SGLT) 2 inhibitors. Over the next 18 months, she experienced multiple hospitalizations for episodes of acute exacerbation of HFpEF, despite compliance with optimal medical therapy, and was noted to have progressively worsening fluid retention.

**Figure 4:**
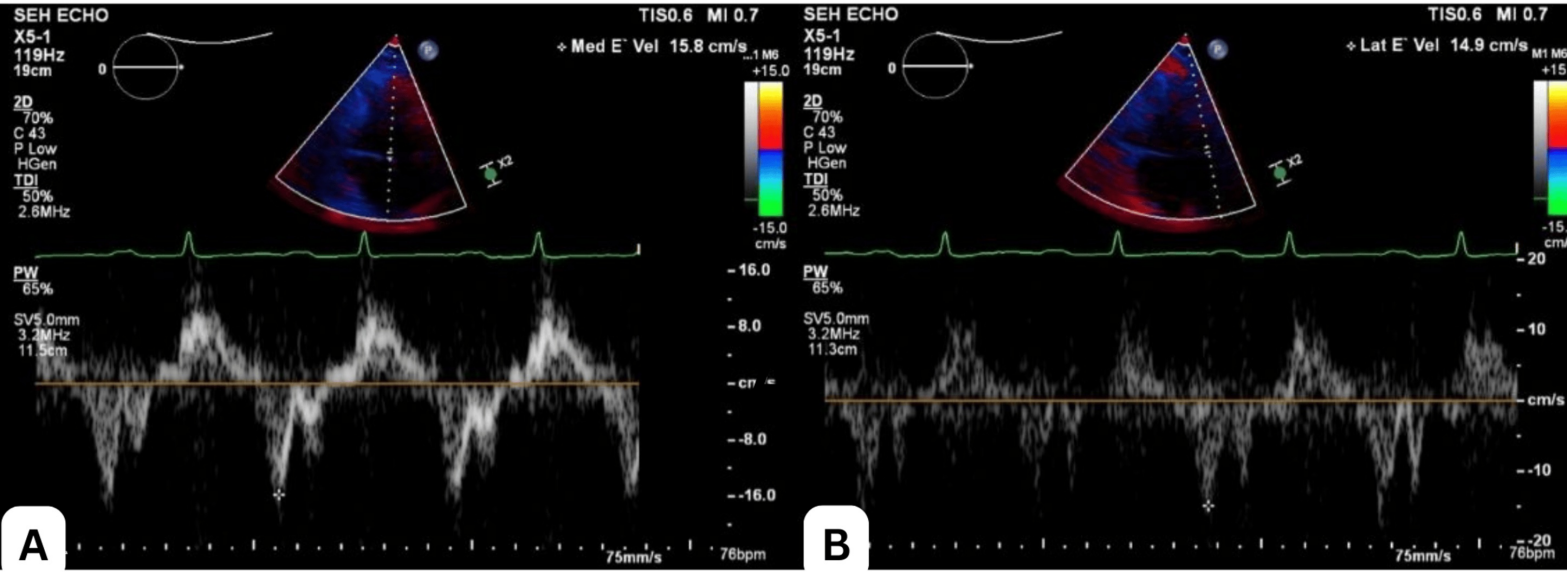
Transthoracic echocardiography (TTE) shows mitral ‘annulus reversus,’ as evidenced by mitral velocity e’ velocity >2% and higher than lateral e’ velocity, which was missed during initial TTE evaluation, the reason being preserved lateral e’ velocity. Figure A shows medial e' velocity of 15.8 cm/s, and Figure B shows lateral e' velocity of 14.9 cm/s.

**Figure 5:**
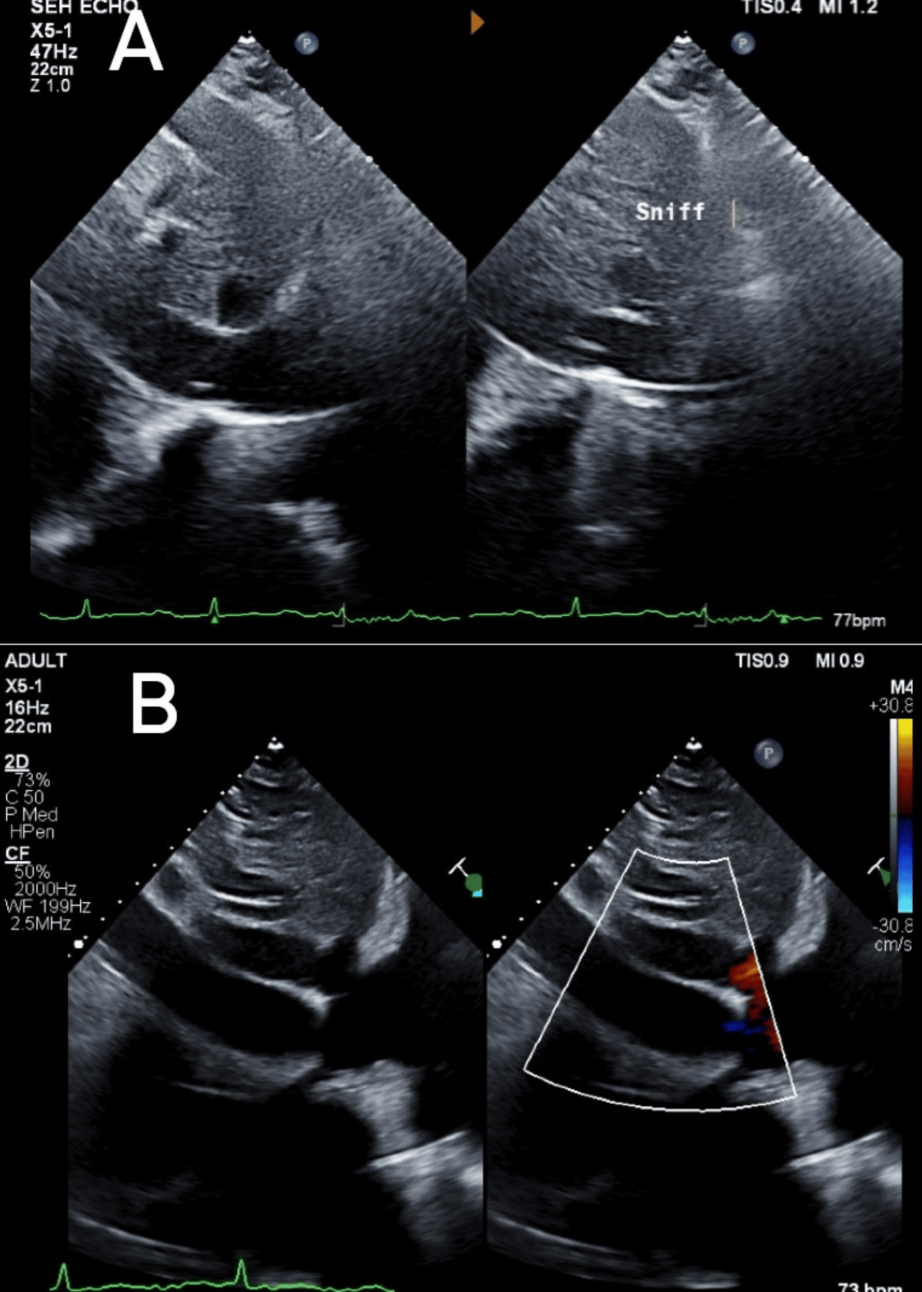
Transthoracic echocardiography in respirophasic cycle. Figure A shows reduced collapsibility of the inferior vena cava (IVC; 2.76 cm) on inspiration. Normally, it's collapsible in inspiration. Normal IVC diameter is 2.9 cm here. Figure B shows a plethoric IVC during inspiration.

**Figure 6:**
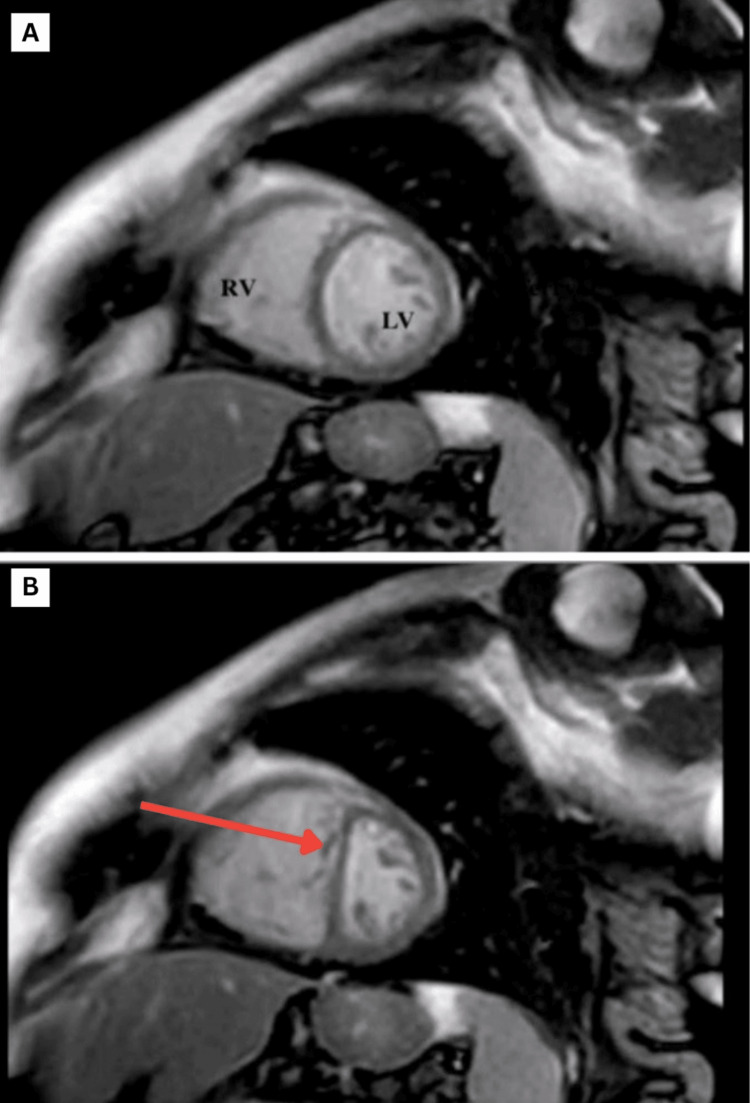
Cardiac magnetic resonance imaging (MRI) Figure A shows a normal interventricular septum in diastole. Figure B shows a leftward shift towards the ventricle known as septal bounce or flattening in inspiration.

A repeat TTE revealed ventricular septal bounce, which is a dynamic echocardiographic finding characterized by abnormal, rapid, and exaggerated motion of the interventricular septum during early diastole, often with a brief posterior and then anterior movement (Video 1); a plethoric inferior vena cava with reduced respiratory collapse (Figure 4); and a positive annulus reversus sign characterized by a reversal of the normal relationship between early diastolic mitral annular velocities: the medial (septal) e′ velocity exceeds the lateral e′ velocity. Normally, the lateral e′ is higher than the medial e′, but in constrictive pericarditis, pericardial tethering of the lateral annulus leads to a reduced lateral e′, while the medial e′ remains preserved or is even increased (Figure 5), suggestive of CP. A review of the previous TTE revealed that the septal bounce had been missed. Hepatic vein flow and respiratory cycle monitoring had not been performed at that time. Right heart catheterization showed mildly elevated pulmonary artery pressure (29 mmHg). Cardiac magnetic resonance imaging (MRI) revealed pericardial thickening, septal bounce, interventricular dependence, and biatrial dilation (Figure 6), confirming the diagnosis of CP.

**Video 1:** Repeat transthoracic echocardiography revealed ventricular septal bounce in early diastole.

She was referred to a higher center for pericardiectomy per her preference. She underwent pericardiectomy and was discharged in stable condition with oral diuretics, SGLT2 inhibitors, and a mineralocorticoid receptor antagonist. On follow-up, the patient reported significantly improved symptoms. Follow-up at six months post-pericardiectomy showed sustained symptomatic improvement to New York Heart Association (NYHA) class I and normalization of right-sided pressures on echocardiography; N-terminal pro-B-type natriuretic peptide (NT-proBNP) levels fell from 1200 to 210 pg/mL.

## Discussion

CP is a rare condition with a prevalence likely underestimated due to frequent misdiagnosis. Globally, tuberculosis remains the leading cause; however, in developed countries, CP is typically idiopathic or secondary to previous viral infections, cardiac surgery, or radiation. While pericarditis can occur in up to 20% of patients with systemic lupus erythematosus, and 30%-50% of RA patients may have asymptomatic pericardial effusions, progression to constrictive physiology is rare, which was presumed to be the etiology for chronic CP in the presented case [7,8]. Clinical manifestations commonly align with those of right-sided heart failure, including elevated jugular venous pressure, ascites, peripheral edema, Kussmaul’s sign, and a pericardial knock. Consequently, CP may clinically overlap with conditions such as restrictive cardiomyopathy, severe tricuspid regurgitation, cirrhosis, or chronic obstructive pulmonary disease (COPD) with cor pulmonale. In contrast to cardiomyopathies like restrictive cardiomyopathy, BNP levels in CP are typically normal or only mildly elevated, as observed in this case [9]. Additionally, inflammatory markers such as ESR and CRP may be elevated, particularly in autoimmune-related CP. 

Echocardiography is the initial imaging modality for diagnosing constrictive pericarditis (CP), and the diagnosis is established based on the Mayo Clinic Diagnostic Criteria, which include respirophasic septal motion bounce, respiratory variation in mitral E velocity (>25%), medial mitral e′ velocity ≥9 cm/s, medial-to-lateral e′ ratio ≥0.91, and hepatic vein expiratory diastolic reversal ratio ≥79% [10]. In our case, medial e′ was 15.8 cm/s, lateral e′ was 14.9 cm/s, and septal bounce was evident, consistent with CP. Although 93% of septal bounce occurrences are associated with CP, the specificity remains low, as septal bounce may also be observed in left bundle branch block (LBBB), right ventricular pacing, cardiac tamponade, or pulmonary hypertension [10].

Multimodality imaging, including CT and cardiac MRI, is often critical for further evaluation of suspected cases. Cardiac MRI has a 93% sensitivity for detecting pericardial thickening greater than 4 mm [11]. However, the absence of thickening does not exclude CP, as some patients may present with constriction without radiographic evidence [12,13]. Cardiac catheterization remains the gold standard diagnostic approach, particularly when noninvasive evaluations are inconclusive. Diagnostic hemodynamic findings for CP include [14,15]: Equalization of right and left ventricular end-diastolic pressures (within 5 mmHg), rapid early diastolic filling with a "dip-and-plateau" or "square root" sign, interventricular dependence (discordant respiratory changes in RV and LV systolic pressures), and dissociation between intrathoracic and intracardiac pressures during respiration.

According to the ACC/American Heart Association (AHA) guidelines, radical (complete) pericardiectomy remains the mainstay of treatment for patients with constrictive pericarditis, particularly those with non-inflammatory disease or those who do not respond to anti-inflammatory therapy for inflammatory pericarditis. In patients with evidence of active inflammation, reflected by elevated inflammatory markers such as ESR or CRP or by cardiac MRI findings (pericardial calcification or absence of active inflammation), an initial trial of anti-inflammatory therapy is recommended for several weeks, with close monitoring for clinical improvement. Postoperatively, patients with idiopathic or viral pericarditis generally have more favorable outcomes. Conversely, individuals with comorbid conditions such as advanced age, renal dysfunction, pulmonary hypertension, or prior radiation exposure, as well as those with underlying systemic inflammatory disease or significant myocardial involvement, tend to experience poorer prognoses [16,17]. Diuretics may also aid in symptomatic management; however, they don't have any mortality benefit. If misdiagnosed or untreated, CP carries a mortality rate exceeding 90% [18]. This case emphasizes the diagnostic challenge posed by CP, particularly given its nonspecific clinical presentation that can closely resemble HFpEF. The unusual association with rheumatoid arthritis further complicates recognition. Clinicians should maintain a high index of suspicion for CP in patients with diuretic-refractory HFpEF phenotypes. Comprehensive echocardiographic assessment, including respiratory variation in transvalvular flows and hepatic vein Doppler analysis, should be pursued early. Integration of multimodal imaging and invasive hemodynamic evaluation via catheterization is essential for definitive diagnosis and timely therapeutic intervention.

## Conclusions

CP is a rare but potentially reversible cause of diastolic heart failure, characterized by pericardial fibrosis and calcification that impair ventricular filling. Advances in imaging, particularly cardiac MRI and hemodynamic assessment via catheterization, have significantly improved diagnostic accuracy, especially in differentiating constriction from restrictive cardiomyopathy. While medical management may offer temporary symptomatic relief in selected inflammatory cases, definitive treatment lies in surgical pericardiectomy, which, when performed timely, is associated with favorable long-term survival and functional recovery. Early recognition and multidisciplinary evaluation are critical to improving patient prognosis and preventing irreversible myocardial damage.
